# Expression in *E. coli *and characterization of the catalytic domain of *Botrytis cinerea *chitin synthase

**DOI:** 10.1186/1756-0500-3-299

**Published:** 2010-11-11

**Authors:** Hervé Magellan, Thierry Drujon, Annie Thellend, Annie Piffeteau, Hubert F Becker

**Affiliations:** 1 UPMC Univ Paris 06, CNRS UMR 7203, Laboratoire des Biomolécules, Paris F-75005, France; 2 et Université Paris Diderot, Paris, France; 3 Laboratoire d'Optique et Biosciences, INSERM U696, CNRS UMR7645, Ecole Polytechnique, Palaiseau, France

## Abstract

**Background:**

Chitin synthase 3a (CHS3a) from *Botrytis cinerea *(Bc) catalyses the multiple transfer of *N*-acetylglucosamine (GlcNAc) residues to the growing chitin chain. Chitin, a β-1,4 linked GlcNAc homopolymer, is an essential cell wall component of filamentous fungi. Chitin synthase, processive membranous protein, has been recognized as a promising target for new antifungicides. Enzymatic characterizations of chitin synthases have been limited, mainly because purity and amounts of integral enzyme obtained after purification procedures have not been sufficient.

**Findings:**

We undertook the preparation of two BcCHS3a fragment proteins, containing only the central domain and devoid of the N-terminal and transmembrane C-terminal regions. The central domain of CHS3a, named SGC (Spsa GntI Core), is conserved in all UDP-glycosyltransferases and it is believed to contain the active site of the enzyme. CHS3a-SGC protein was totally expressed as inclusion bodies in *Escherichia coli*. We performed recombinant CHS3a-SGC purification in denaturing conditions, followed by a refolding step. Although circular dichroism spectra clearly exhibited secondary structures of renatured CHS3a-SGC, no chitin synthase activity was detected. Nevertheless CHS3a-SGC proteins show specific binding for the substrate UDP-GlcNAc with a dissociation constant similar to the Michaelis constant and a major contribution of the uracil moiety for recognition was confirmed.

**Conclusions:**

Milligram-scale quantities of CHS3a-SGC protein with native-like properties such as specific substrate UDP-GlcNAc binding could be easily obtained. These results are encouraging for subsequent heterologous expression of full-length CHS3a.

## Background

Chitin, a linear β-(1-4)-linked polymer of *N*-acetylglucosamine (GlcNAc), is an important structural component of the cell walls of many fungi, with different contents of chitin between species [1]. Chitin is absent from plant and mammalian species. Thus, its biosynthesis is recognized as a valuable and attractive target for the design of fungicides [2]. Chitin is synthesized by chitin synthases (CHS), a multiple membrane isoenzyme family. Analysis of several fungal genomes allowed the classification of yeast CHS, *Saccharomyces cerevisiae *and *Candida albicans*, into three classes and those of many filamentous fungi into seven or more classes [3,4]. Results were based on the identification of a conserved domain present in all CHS described. For *Botrytis cinerea *(Bc), a necrotrophic filamentous fungus which causes grey mould on a wide range of plants, eight distinct CHS genes were isolated and classified into two divisions and seven classes based on the homology of amino acid sequences and topology [5]. More generally CHS are processive glycosyltransferases capable of successively transferring a number of GlcNAc monomers from a UDP-GlcNAc donor to the reducing end of a growing acceptor polymer [6]. A number of fungicides have been used to control plant contamination by *Botrytis cinerea*, but serious resistance problems have arisen [7]. Chitin biosynthesis as antifungal target presents a new strategy for effective control of *Botrytis cinerea*. Among different CHS mutants, disruption of chitin synthase class IIIa gene (BcCHS3a) in the *Botrytis cinerea *genome presented interesting results. The resulting mutant exhibited a reduction in chitin content and a reduction of *in vitro *chitin synthase activity, compared with the wild-type strain. Moreover, BcCHS3a mutant had a largely reduced virulence on plant [8,9]. Taking into account these results, BcCHS3a enzyme was chosen for further characterization.

**Figure 1:**
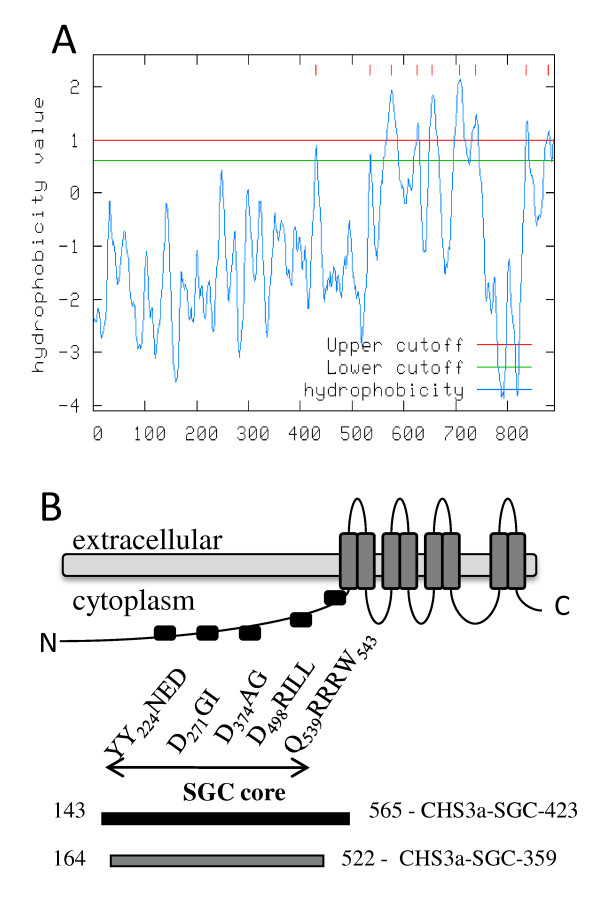
**Transmembrane protein topology of *Botrytis cinerea* CHS3a. **(A) Hydropathy profile of BcCHS3a based on the protein sequence, determined on http://mobyle.pasteur.fr web site by using the "Toppred" program. Hydrophobicity values above the cutoff correspond to potential transmembrane domains. Results supposed eight transmembrane domains at the C-terminal region of the protein. (B) Schematic representation of wild type CHS3a and recombinant proteins fragments. Predicted transmembrane domains and conserved sequence motifs in BcCHS3a obtained after HCA plots of BcCHS3a sequence with other non processive glycosyltransferase sequences [5]. CHS3a-SGC-423 (143-565) is indicated in black and CHS3a-SGC-359 (164-522) in grey below the graph.

CHS3a is a 916 amino acid protein with eight putative membrane regions occuring mainly in the C-terminus region (Figure 1A). Prediction of transmembrane domains was performed on the "Mobyle Institut Pasteur" web site, by using the "Toppred" program which is a transmembrane topology predictor in protein sequences http://mobyle.pasteur.fr. CHS3a belongs to the "SGC" UDP-GlcNAc-β-glycosyltransferase family consisting of proteins with no significant sequence identity, but which all contain a structurally very similar catalytic domain [10]. These enzymes share the same catalytic function, transferring the same substrate UDP-GlcNAc onto different acceptors. It is very likely that SGC domain of CHS3a contains the active site of chitin synthase. This domain was named SGC for SpsA GnT I Core and was deduced from crystal structures from the two nonprocessive β-glycosyltransferases SpsA - *Bacillus subtilis *and GnT I - rabbit ([11-13] for review). Hydrophobic cluster analysis (HCA) [14] of the CHS3 amino acid sequence with SpSA and GnT1 sequences revealed one conserved tyrosine and three conserved aspartate for the CHS3-SGC domain [5] (Figure 1B). Y224 (motif TYYNED), D271 (motif DGI) and D374 (motif DAG = motif DXD are thought to be implicated in substrate binding, whereas the third aspartate D498 (motif LAEDRIL) is probably the catalytic base [5]. Downstream of the SGC domain two other conserved sequences, QRRRW and WG, were identified in all processive polysaccharide synthases, but these were absent from non-processive glycosyltransferases. QRRRW and WG motifs were probably implicated in the processivity of the polysaccharide synthases (Figure 1B). Previous work on *Saccharomyces cerevisiae *chitin synthase 2 has demonstrated that to retain chitin synthase activity, no conserved amino acid mutation in the highly conserved region, corresponding to the SGC domain, is allowed [15]. The SGC domain seems essential for sugar transfer from UDP-sugar to an acceptor, and the transmembrane domains seem implicated in the multiplicity of GlcNAc transfer to an acceptor.

To our knowledge, no active recombinant chitin synthase has been successfully expressed and purified in large amounts for a complete enzymatic characterization. Nevertheless, several previous works revealed common biochemical properties of CHSs, such as their cellular localization in the plasma membrane, their description as zymogenic enzymes, their possible allosteric regulation by N-acetyl-glucosamine and their divalent cation stimulating effect on enzymatic activity ([16-19]). It seems very difficult to express active CHS in large amounts because of their large molecular mass and their transmembrane association. Recently, two new attempts have been made to purify the active chitin synthase of *Wangiella dermatitidis *[20] and chitin synthase from the midgut of *Manduca sexta *[21] by immunoaffinity purification. In both cases, as in the previous attempts, the amount of purified enzyme limited further enzymatic characterization of CHS.

Previously, CHS2 of *Saccharomyces cerevisiae *characterization revealed that i) the N-terminally truncated CHS2 of *S. cerevisiae *exhibits the same enzymatic activity as full size enzyme and ii) the 35 kDa fragment corresponding to the region just before the first transmembrane domain should contain the active site of the enzyme [22]. By taking these results into account, it is conceivable that a small part of CHS, corresponding to the SGC domain previously described, could be sufficient for catalytic activity, while other domains of the enzyme are implicated in other functions, such as membrane localization, binding to chitin and export of chitin fibers.

To investigate the enzymatic properties of chitin synthase, cloning and expression in *E. coli *of the BcCHS3a recombinant protein including only SGC domain, devoid of both the non-conserved N-terminus region and the highly hydrophobic transmembrane C-terminus region, called CHS3a-SGC-423, was undertaken. CHS3a-SGC-359, a shorter C-terminal truncated CHS3a-SGC form, with additional deletion of both the conserved QRRRW motif and a few residual hydrophobic amino acids, was also prepared. The purification, folding, enzymatic activity and UDP-GlcNAc binding of both CHS3a fragments were investigated.

## Methods

### Vector construction

*Botrytis cinerea *(BD90 strain) CHS3a cDNA was prepared as previously described [5]. The CHS3-SGC-423 sequence was excised by PCR amplification using forward (5'-GCTAGCGCGTACTCTGGAAACGGAGGC-3') and reverse (5'-TTAACGTCTCATGAAGCAGCACATGATACC) primers. The forward primer was engineered to contain a single NheI site (underlined). The PCR product was purified on agarose gel before ligation into a pGEM-T Easy Vector by AT cloning (Promega) and transformed into DH5α cells for colony screeening and plasmid purification (pGEM-T Easy Vector SystemI, Promega). To express protein in *E. coli*, CHS3-423 sequence was excised from pGEM-T Easy Vector using NheI and SacI cleavage and cloned, with T4 DNA ligase, into pET-28a(+) vector (Promega), previously cut by the same restriction enzymes. The pET-28a:CHS3a-SGC-423 construct was transformed in *E. coli *DH5α cells. Positive clones were selected and the plasmid extracted and purified (Wizard Plus Minipreps, Promega). Sequences of the resultant constructs were checked by DNA sequencing (Millegen, Labège, France). The resulting plasmid pET-28a:CHS3a-SGC-423 transformed in *E. coli *BL21(DE3) encodes a fusion protein designated CHS3a-SGC-423, which consists of a His_6_-Tag at the N-terminus followed by a 423 amino acids sequence of the central domain of CHS3 (from residues 143 to 565).

Truncated CHS3-SGC-359 gene was cloned in a pET-30 Ek/LIC vector (ligation-independant cloning system, Novagen). The CHS3-SGC-359 sequence was excised from pET-28a:CHS3a-SGC-423 as described above with 5'-GACGACGACAAGATCAAAAATGCAATTCAG-3' and 5'-GAGGAGAAGCCCGGTTTATTTAGCTGCCTT-3' primers. pET-30:CHS3a-SGC-359 plasmid encodes for CHS3a-SGC-359 protein, which consists of a His_6_-Tag at the N-terminus followed by a 359 amino acids sequence of the central domain of CHS3, from residues 164 to 522.

### Protein expression

pET-28a:CHS3a-SGC-423 and pET-30:CHS3a-SGC-359 expression vectors were transformed into competent BL21(DE3) cells. An overnight starter culture was established in Luria-Bertani (LB) medium (casein peptone plus 10 g/l, bacto yeast exctract 5 g/L, NaCl 10 g/l) supplemented with kanamycin (50 μg/ml). The next morning 8 mL of the culture was added to 800 ml of LB media supplemented with 50 μg/ml kanamycin. The production culture was grown with shaking (145 RPM) at 37°C until an absorbance at 600 nm around 0.7 was reached, whereupon expression of recombinant CHS3a was induced by addition of 1 mM IPTG (isopropyl β-D-thiogalactopyranoside). Cells were then allowed to grow for a further one night for CHS3a-SGC-423 or 3 h for CHS3a-SGC-359 before being harvested by centrifugation at 5000 × g at 4°C for 20 min. Cells pellets were frozen at -20°C.

### CHS3-SGC-423/359 extraction from urea-solubilized inclusion bodies

Cell pellets were resuspended in 15 ml cold 20 mM phosphate buffer pH 8, containing Triton 0.1%, 20 mM NaCl, 2 μM leupetin, 2 μM pepstatin and 2 mM alpha-toluenesulfonyl fluoride (buffer A) and disrupted by ultrasonic treatment (10 times 15 sec). The crude homogenate was centrifuged at 5000 × g for 15 min at 4°C and solubilization of the inclusion body pellet was achieved in 15 ml cold buffer A containing 8 M urea. The solution was incubated overnight at 4°C with shaking. To remove insoluble material, the solution was centrifuged at 5000 × g for 20 min and the supernatant, containing recombinant protein, was used for purification.

### Purification and refolding procedure

#### Nickel affinity chromatography for CHS3-SGC-423

Urea-denaturated His6-tagged CHS3a was applied to a 1 mL Ni^2+^-HiTrap chelating HP column (Amersham Biosciences). The column was operated at a flow rate of 1 ml/min. Previously the column was loaded with Ni^2+ ^with a solution of NiSO_4_,6 H_2_O (1 ml; 0.3 M) and equilibrated with 20 mM phosphate buffer pH 7.5, containing 500 mM NaCl, 6 M urea and 0.01% CHAPS. Protein elution was performed using a linear gradient of 0-500 mM imidazole in the same buffer over 30 min. The His6-tagged CHS3 proteins were eluted between 100 and 200 mM imidazole.

#### Carboxymethyl ion exchange chromatography for CHS3-SGC-423

Recovered fractions were equilibrated in a new buffer (20 mM phosphate buffer pH 6.5, 6 M urea and 0.01% CHAPS) using a PD-10 column (Amersham Biosciences) before further purification to homogeneity was performed. Fractions were applied to a carboxymethyl column (1 mL, Amersham Biosciences), His6-tagged CHS3 proteins fixed on the column were subjected to a refolding step by a linear gradient of urea from 6 to 0 M in a total volume of 30 ml. The recombinant protein was eluted with a linear gradient of NaCl from 0 to 500 mM in a total volume of 30 mL.

#### Two successive nickel affinity chromatographies for CHS3-SGC-359

The first nickel affinity chromatography was performed in the same conditions described above for CHS3-SGC-423. The second purification step and refolding procedure were also performed on a nickel affinity column. The conditions were as follows: nickel affinity column loading buffer (20 mM phosphate buffer pH 7.5, containing 6 M urea, 0.1 M NaCl, 0.01% CHAPS and 0.1 mM DTT), refolding step by a linear gradient of urea from 6 to 0 M in a total volume of 30 ml. The refolded recombinant protein was eluted with a linear gradient from 0 to 100% of elution buffer (20 mM phosphate buffer pH 7.5, 0.5 M NaCl, 0.5 M Imidazole, 0.01% CHAPS and 0.1 mM DTT).

### Circular Dichroism (CD)

Measurements were carried out with a JASCO 840 spectropolarimeter using a 1 mm path length cell. The displayed CD spectra were obtained at 25°C from the average values for 3 scans between 200 nm and 350 nm on 300 μl of protein fraction at 4 μM, with the signal being corrected for background using the protein buffer solution. Both native and denatured proteins were analyzed. Data are presented in units of [θ] (deg.cm^2^.mol^-1^). Secondary structure was predicted by applying the Dichroprot procedure http://dicroprot-pbil.ibcp.fr/[23].

### Chitin synthase assay

CHS3-SGC-423 (2 μg, 1 μM) was added to a standard reaction mixture (in a total volume of 50 μl: digitonin (5 μl, 2%), magnesium acetate (5 μl, 50 mM), trypsin (5 μl, 0,2 mg/mL, 10000 units/mg protein, Sigma), *N*-acetylglucosamine (2 μl,1 M), UDP-*N*-acetylglucosamine (2.5 μl, 10 mM), UDP-*N*-acetyl[^14^C]glucosamine (1 μl, 10 nCi, 288 mCi/mmol) in Tris-HCl buffer 25 mM pH = 7.4). Incubation was carried out at 30°C for 10 min until the reaction was stopped with 1 ml of 10% trichloroacetic acid. The radioactivity in the insoluble fraction was counted after filtration through glass-fiber filter (Whatman, GF/C). The soluble fraction was discarded as it contained only unincorporated UDP-*N*-acetyl[^14^C]glucosamine. The filter was washed with 1 M acetic acid/ethanol : 30/70, dried and radioactivity measured in a liquid scintillation counter (LKB 1214 RackBeta).

### Fluorescence spectroscopy

Fluorescence titrations were conducted using a Spectrofluorimeter PTI (Photon Technology International) at 25°C using an excitation wavelength of 290 nm and monitoring emission between 300 and 400 nm at a scan rate of 1 nm/sec. Solutions of UDP-GlcNAc (0 - 8 mM) and CHS3-SGC-423 (1 μM) were allowed to equilibrate in 20 mM potassium phosphate, pH 6.5 and Mg(OAc)_2 _10 mM under continuous stirring for a few minutes prior to recording each spectrum. The fluorescence quenching was analyzed by fitting data to (1) (y/ymax) = [L]/(Kd+[L]) where y/ymax equals the fraction quenched (1 - F/F0), F0 and F are the maximum observed relative fluorescence intensities at the emission wavelength 340 nm before and after substrate addition, respectively. [L] is the concentration of unbound UDP-GlcNAc and Kd is the concentration of substrate giving half-maximal quenching, which is assumed to be identical to the dissociation constant, Kd, of the respective substrate. The fitting parameters were Kd and Ymax; Ymax equals the fraction quenched at infinite substrate concentration.

### Results and discussion

#### Preparation of pET28-CHS3a-SGC-423 *E. coli *expression vector and CHS3a-SGC-423 protein expression

**Figure 2:**
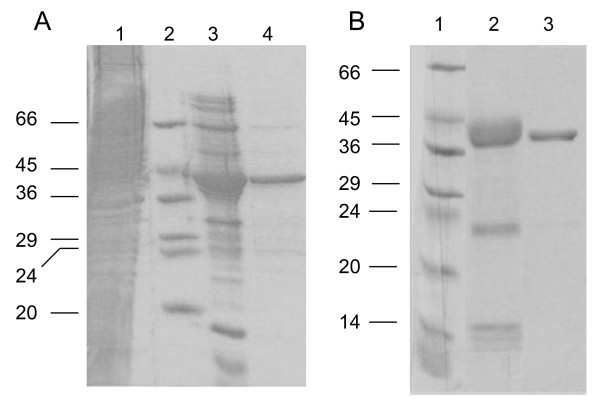
**Sodium dodecyl sulfate polyacrylamide gel electrophoresis (SDS-PAGE) analysis of protein samples from *E. coli *BL21(DE3) cells transformed by pet28-CHS3a-SGC-423 (A) or pet30-CHS3a-SGC-359 (B) plasmids**. Aliquots of successive steps of purification : (A) CHS3a-SGC-423 Lanes : 1; whole cell fraction after solubilization of inclusion bodies; 2, molecular weight markers; 3, Ni^2+^-NTA-agarose column; 4, carboxymethyl-agarose column. (B) CHS3a-SGC-359 Lanes 1, molecular weight markers; 2 and 3, first and second Ni^2+^-NTA-agarose columns, respectively. The electrophoresis was performed in 10% PAGE and gels were stained by Coomassie blue.

The primary structure analyses of BcCHS3 revealed that the minimal SGC domain (residues 143 to 565) containing all conserved residues and preserving intact secondary structures on each extremity, corresponds to a protein of 423 residues, named CHS3a-SGC-423. Amplification of the corresponding gene was performed from *B. cinerea *RNA by RT-PCR with appropriate primers, subcloned into pGEM-T Easy Vector and transfered into a prokaryotic expression vector (pET28a). *B. cinerea *CHS3a-SGC-423 protein was produced in the *E. coli *strain BL21(DE3) transformed with pET28-CHS3a-SGC-423 vector to produce a fusion protein containing an *N*-terminal His-Tag. The recombinant CHS3a-SGC-423 protein was expressed mainly as insoluble inclusion bodies. Decreasing the induction temperature or IPTG concentration did not enhance the solubility of the recombinant protein. CHS3a-SGC-423 protein was solubilized in denaturing buffer and purified, under denaturing conditions, by Ni^2+^-NTA affinity chromatography and eluted with imidazole gradient buffers (Figure 2A, lane 3). After buffer exchange, fractions containing recombinant protein at pH 6.5 were loaded on a carboxymethyl-agarose column. Protein refolding took place on the column before elution and led to a purified CHS3a-SGC-423 protein (Figure 2A, lane 4) with a yield of 0.7 mg per liter of culture.

### Renaturation and structural analysis of BcCHS3a-SGC-423

As most of the overexpressed protein was present in the insoluble fraction, correct protein refolding was essential. Denatured protein was immobilized on a carboxymethyl-agarose matrix and subsequent denaturant dilution to promote refolding was performed. Refolded CHS3a-SGC-423 was eluted with a NaCl gradient.

**Figure 3:**
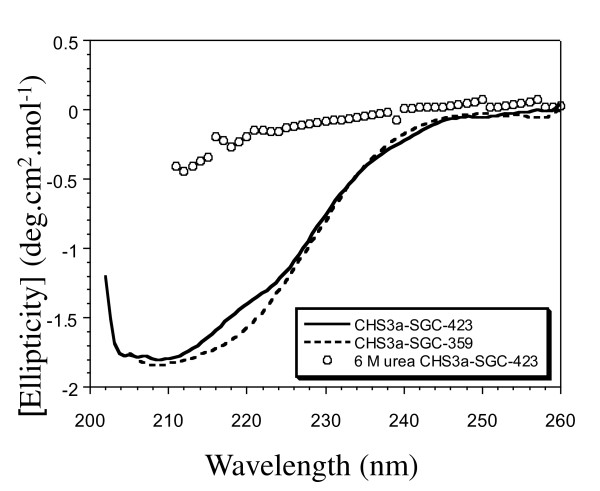
**Circular dichroism analysis of purified CHS3a proteins fragments**. Far-UV spectra of CHS3a-SGC-423 in completely denatured state (circles) and refolded state at pH 7.5 (black continuous line) and spectra of CHS3a-SGC-359 in refolded state at pH 7.5 (black dotted line).

The structural integrity of the recombinant protein was further confirmed by circular dichroism (CD) spectral analysis. The plot of mean residue molar ellipticity of refolded CHS3-SGC-423 is considerably different from denaturated protein and clearly exhibits secondary structure formation (Figure 3). Curve fitting analysis of the CD spectrum revealed that CHS3a-SGC-423 is composed of 16% α-helix, and 15% β-sheets. In the absence of a BcCHS3a structural model, results could only be compared to secondary structure prediction deduced from the amino acid composition of CHS3a-SGC-423 (36% α-helix, 12% β-sheets). These results indicated that the CHS3a-SGC-423 protein contains significant refolded elements following this treatment.

### Chitin synthase assays

Enzymatic activity of CHS3a-SGC-423 protein was assayed by measuring the rate of formation of ^14^C-chitin from UDP-^14^C-GlcNAc [24], in presence of Mg^2+ ^and GlcNAc. Mg^2+ ^has been described as an essential cation for substrate binding in the active site, and GlcNAc is a known allosteric effector for chitin synthase activity [18,17]. Contrary to the classical test described by Orlean and co-workers [24], trypsin addition was omitted since recombinant CHS3a-SGC-423 was not produced as a zymogenic form and thus did not require preliminary trypsin activation. Using these conditions, no chitin synthase activity was detected. As CHS3a-SGC-423, devoid of a transmembrane C-terminal domain, did not show chitin synthase acivity, we can suppose that the transmembrane domains are implicated in the membrane localization of the protein and as a result, involved in substrate binding and/or the catalysis reaction step.

### Protein-substrate complex formation measured by tryptophan fluorescence

**Figure 4:**
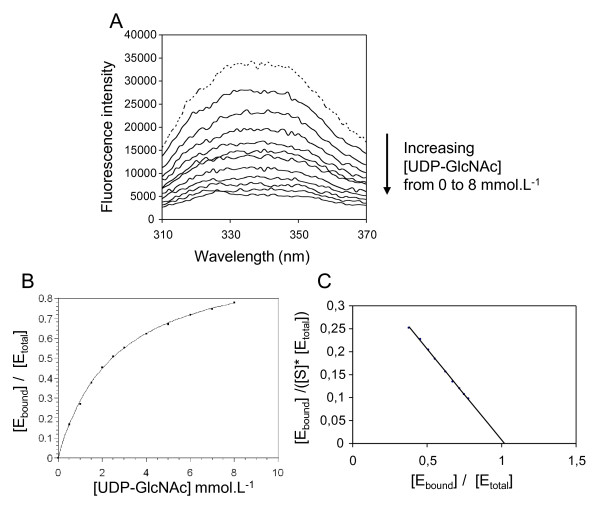
**Effect of UDP-GlcNAC on the intrinsic tryptophan fluorescence of CHS3a-SGC-423 protein and Binding Profiles.** (A) Emission spectra of 1 μM CHS3a-SGC-423 incubated in 20 mM potassium phosphate, pH 6.5, 10 mM MgOAc with increasing concentrations of UDP-GlcNAC (0.5, 1, 2, 2.5, 3, 4, 5, 6, 7, and 8 mmol.L^-1^) from the top to the bottom (continuous lines). Reference spectrum for CHS3a-SGC-423 without substrate shown with a dotted line. Maximal fluorescence value « F » at 340 nm was used to quantify bound proteins, « F_0 _» corresponds to the unbound protein signal. (B) Saturation curve [E_bound_]/[E_total_] = f([UDP-GlcNAc]) presents an hyperbolic profile. [E_bound_]/[E_total_] was obtained from (1 - F/F0) point at 340 nm for each curve in panel A. (C) Scatchard plot of UDP-GlcNAc specific binding to CHS3a-SGC-423. [E_bound_]/([S]*[E_total_]) mmol^-1^.L versus [E_bound_]/[E_total_] was plotted.

To determine if the C-terminal transmembrane domain of CHS was essential for substrate binding, we investigated the formation of a stable enzyme-substrate complex between renaturated CHS3a-SGC-423 and UDP-GlcNAc. Fluorescence spectroscopy of the intrinsic Trp residues of CHS3a-SGC-423 was ideally suited for monitoring the conformational changes in the Trp microenvironments that may accompany the binding of the substrate. Emission spectra of the free wild-type CHS3-SGC-423 and in the presence of increasing concentrations of UDP-GlcNAc, when excited at 290 nm are shown in Figure 4A. The fluorescence spectra are in agreement with previously reported Trp spectra and exhibited a classic maximum emission at 340 nm when excited at 290 nm [25]. By close interaction between the protein and the substrate, the fluorescence spectrum of CHS3a-SGC-423 exhibited a change in intensity for maximum emission and displayed a substrate concentration dependance (Figure 4A). A re-plot of the quenching effect versus UDP-GlcNAc concentration revealed saturating curves by fitting the data to equation (1) (Figure 4B). The resulting Kd is 2.6 mmol.L^-1 ^± 0.2 mmol.L^-1 ^for UDP-GlcNAc (Table 1), which is of the same order of magnitude as the Michaelis constant, Km, of 2.4 mmol.L^-1^, for *B. cinerea *CHS for UDP-GlcNAc [26]. The SGC domain of CHS3a contains six Trp residues occuring at positions 254, 261, 356, 388, 513 and 541. Thus, the fluorescence changes observed for the binding of UDP-GlcNAc were difficult to assign to one specific Trp. Nevertheless, at least one Trp appeared to be in close proximity to the UDP-GlcNAc binding site.

**Table 1:** Kd _UDP-GlcNAc _for CHS3a-SGC proteins.

Protein	**Kd UDP-GlcNAc mmol.L**^-1^
CHS3a-SGC-423	2.6 ± 0.2
CHS3a-SGC-359	7.2 ± 0.6

The values ± SD (standard deviation) are representative of 4 independent experiments.

Various UDP-GlcNAc analogs, such as UDP-Glucose, GlcNAc, UTP or ATP were tested. The results showed that CHS3a-SGC-423 preferred a UDP variant rather than UTP or ATP and the highest affinity was reached when UDP carried a carbohydrate moiety (Table 2). The binding of CHS3a-SGC-423 by UDP-GlcNAc may be interpreted as a result of a specific recognition of the nucleotide moiety by the enzyme. This is in agreement with previous results on polyoxin as CHS inhibitor, describing a specific binding site on CHS corresponding to the uridine moiety of UDP-GlcNAc [27]. Furthermore a Scatchard plot determined a stoichiometry of 1:1 between CHS3a-SGC-423 and UDP-GlcNAc in the complex (Figure 4C). The effect of UDP-GlcNAc on the CHS3a-SGC-423 intrinsic Trp fluorescence was attributed to a specific interaction of CHS3a-SGC-423 with UDP-GlcNAc.

**Table 2:** CHS3a-SGC-423 specificity for different ligands binding.

Kd (mmol.L^-1^)				
UDP-GlcNAc	UDP-Glc	UTP	ATP	GlcNAc
2.6 ± 0.2	2.6 ± 0.3	5.3 ± 0.8	21.5 ± 1.7	-

The values ± SD are representative of 3 independent experiments.

"-" = not determined

These results led us to prepare a truncated form of CHS3-SGC-423 by deletion of about forty C-terminal residues to obtain CHS3a-SGC-359. The deletion of a few hydrophobic residues including the conserved QRRRW motif located at the beginning of the first transmembrane domain was expected to yield a soluble form of CHS3a-SGC-359 thus obviating the delicate renaturation step.

### Protein-substrate complex with CHS3-SGC-359 protein

Unfortunately the CHS3a-SGC-359 protein, corresponding to residues 164 to 522, was still expressed as insoluble inclusion bodies in *E. coli*. CHS3a-SGC-359 was purified by two successive Ni^2+^-NTA-agarose affinity columns in presence of 6 M urea, with a protein refolding step on the second Ni^2+^-NTA-agarose column (Figure 2B lanes 2 and 3). CHS3a-SGC-359 was obtained with a high yield of 12 mg of protein for 1L culture and refolding was performed and verified by CD as for CHS3a-SGC-423.

The Kd of the UDP-GlcNAc/CHS3a-SGC-359 complex was determined by fluorescence titration as described above. Interestingly, the Kd of CHS3a-SGC-359 was about three fold higher than for CHS3-SGC-423 upon UDP-GlcNAc binding (Table 1). CD spectra showed a significant difference from 215 to 225 nm between the CHS3a-SGC-423 and CHS3a-SGC-359 proteins (Figure 3). The presence of a longer C-terminal domain containing the QRRRW motif in recombinant CHS3-SGC-423 increased the affinity for UDP-GlcNAc, possibly because this longer C-terminal domain could have a number of different effects on the local or overall folding. Results of the CD spectra are in agreement with this hypothesis. We can however note that he QRRRW motif, which is a signature sequence for polysaccharide synthases, could be one element which participates in the improvement of the protein folding process.

Although recombinant CHS3a containing only the SGC domain of CHS3a does not exhibit chitin synthase activity, specific binding of the UDP-GlcNAc substrate was clearly demonstrated and a higher affinity for the longer C-terminal SGC form was obtained. The recombinant CHS3 protein domains were obtained in large amounts and they displayed plausible binding site folding for substrate recognition. These results are encouraging for the preparation of full size recombinant chitin synthase, including the transmembrane domains for the correct insertion of the protein in the plasmic membrane, possibly a prerequisite for efficient enzymatic activity. Heterologous expression of an active, nonfungal CHS from *Entamoeba histolytica *(EhCHS) was described in *Sacharomyces cerevisiae*. This may be a promising starting point for BcCHS3a heterologous expression, although the overexpressed-EhCHS protein differed considerably from fungal CHS and presented different activity characteristics such as the chitin polymer eviction from the cell wall [28]. In the future, we plan to clone and express the BcCHS3a gene in *Sacharomyces cerevisiae *to produce enough protein to permit an enzymatic assay for the screening of new antifungicides and for elucidating the catalytic properties of chitin synthases.

## Competing interests

The authors declare that they have no competing interests.

## Authors' contributions

HM performed most of the experiments and TD carried out the activity assays. AP assisted with the enzymatic analysis, and drafted portions of the manuscript. AT participated in the interpretation of data and drafted portions of the manuscript. HFB designed and coordinated the experiments, wrote the manuscript and edited the final text that all authors read and approved.
